# Solid-Contact Potentiometric Sensors and Multisensors Based on Polyaniline and Thiacalixarene Receptors for the Analysis of Some Beverages and Alcoholic Drinks

**DOI:** 10.3389/fchem.2018.00134

**Published:** 2018-04-24

**Authors:** Michail Sorvin, Svetlana Belyakova, Ivan Stoikov, Rezeda Shamagsumova, Gennady Evtugyn

**Affiliations:** ^1^Analytical Chemistry Department, A.M. Butlerov' Chemistry Institute, Kazan Federal University, Kazan, Russia; ^2^Organic Chemistry Department, A.M. Butlerov' Chemistry Institute, Kazan Federal University, Kazan, Russia

**Keywords:** solid-contact sensors, potentiometric sensors, electronic tongue, polyaniline, food analysis

## Abstract

Electronic tongue is a sensor array that aims to discriminate and analyze complex media like food and beverages on the base of chemometrics approaches for data mining and pattern recognition. In this review, the concept of electronic tongue comprising of solid-contact potentiometric sensors with polyaniline and thacalix[4]arene derivatives is described. The electrochemical reactions of polyaniline as a background of solid-contact sensors and the characteristics of thiacalixarenes and pillararenes as neutral ionophores are briefly considered. The electronic tongue systems described were successfully applied for assessment of fruit juices, green tea, beer, and alcoholic drinks They were classified in accordance with the origination, brands and styles. Variation of the sensor response resulted from the reactions between Fe(III) ions added and sample components, i.e., antioxidants and complexing agents. The use of principal component analysis and discriminant analysis is shown for multisensor signal treatment and visualization. The discrimination conditions can be optimized by variation of the ionophores, Fe(III) concentration, and sample dilution. The results obtained were compared with other electronic tongue systems reported for the same subjects.

## Introduction

Multicomponent analysis of liquid samples is one of frequently mentioned challenges of modern analytical chemistry. The determination of surface water contamination, drinking water hardness, ionic content of biological fluids, and nutritive value of foodstuffs require simultaneous determination of numerous ionic and neutral species that are naturally typical for the subject of the analysis or occasionally introduced due to contamination (Cuartero et al., 2017). Although most of the problems mentioned are successfully solved by conventional analytical equipment like ionic and high-performance liquid chromatography (Colenutt and Trenchard, 1985), some aspects of the multicomponent analysis of liquids remain in the scope of intensive investigations. Among others, two challenges call for further consideration, i.e., (i) the improvement of existing approaches in terms of higher selectivity of the analysis, and (ii) adaptation of conventional techniques to their application outside chemical laboratory. The on-site (on-demand) application concept assumes the development of portable equipment utilizing chemical sensors with minimal sample pre-treatment and fast and reliable data processing. However, the use of individual sensors is complicated by their non-ideal selectivity toward certain analytes and unexpected influence of interferences, which existence in the sample cannot be reliably predicted. Together with a large number of analytes, this limits the application of chemical sensors to a few most important species present in the sample in moderately high quantities. The limitations mentioned can be overcame by assembling the sensor array, i.e., set of sensors with cross-reactivity/selectivity toward various sample components (Bratov et al., 2010). Their signals are processed using pattern recognition algorithms that extract information on the content of the sample or on its quality, e.g., brand name of foodstuffs (Haddi et al., 2014) or relative value of hot spirits or fresh juices (Tahara and Toko, 2013). Similar systems are utilized for estimation of taste (Ha et al., 2015), antioxidant properties (Kraujalyt et al., 2015), food adulteration (Peris and Escuder-Gilabert, 2016) and drug bitterness (Yaroshenko et al., 2016). Previous applications of sensor arrays in food analysis are reviewed in Escuder-Gilabert and Peris (2010).

Among various sensor arrays, potentiometric sensors based on ion-selective electrodes (ISEs) show undisputable advantages, e.g., fast and reversible response, sufficient reproducibility, simple measurement protocol, compatibility with portable ionometers, well elaborated theory and intuitively understandable principles of signal interpretation. In 1991–1993, C. Di Natale and Yu. Vlasov have proposed the arrays of potentiometric sensors that were defined together with the algorithms of the signal processing as “Electronic Tongue” (ET) (Di Natale et al., 1996). Since then, potentiometric ETs have been applied for classification and quantitative analysis of biological fluids (Gutiérrez et al., 2007), foodstuffs (Beullens et al., 2008), medicines (Legin et al., 2004), waste, and natural waters (Di Natale et al., 1997; Mimendia et al., 2010) and so on.

The efficiency of multisensor systems depends on performance of individual sensors. High stability of the response and variable selectivity toward various species promote discrimination of the analytes and assessment of the complex samples in accordance with quality parameters. Meanwhile previous history of ISEs was directed to the achievement of maximal selectivity of the response toward individual analytes. The potentiometric sensor arrays first involved conventional ISEs based on glass and chalcogenide glass membranes (Di Natale et al., 1996). Such an approach resulted in necessity of too many individual ISEs to establish necessary cross-selectivity and discriminate ionic species present in the sample. Later on, the ISEs with polymeric membranes containing different ionophores have been introduced to extend the performance of the multisensor systems. Porphyrin derivatives (Paolesse et al., 2003), either free or bonded to transient metal ions, were mainly applied in the potentiometric ETs. Then, conventional polymeric membranes and ionophores were substituted by semiconductor devices, e.g., field effect transistors (Ipatov et al., 2009), and planar metal electrodes directly contacted with the membranes (Ciosek et al., 2007). Many of the ETs described were applied also in flow-through mode (Wesoły et al., 2016).

Despite obvious progress in the development of sensors and software for the ET assembling and data processing, there is a high interest to the further enhancement of the number of ionophores and to the improvement of the ISE's design. This interest is mainly related to the drawbacks of current ET systems, i.e., rather large number of individual sensors, significant correlation of their signals and limited number of problems to be solved with ET systems. Multi-purpose ET systems with lower number of ISEs will simplify their operation outside chemical laboratory and accelerate data processing based on statistical methods of data mining and pattern recognition.

In this respect, the use of macrocyclic ionophores together with polyaniline as a platform for solid-contact potentiometric sensors is very promising in the ET systems design due to the compact and easily machining design and prospects of tuning both selectivity and sensitivity of response toward different species and complex media. In this review, own and literary data related to the use of polyaniline sensors with macrocyclic ionophores in the analysis of foodstuffs and beverages are summarized.

## Polyaniline in the assembly of potentiometric sensors

Polyaniline is the polymer that can be obtained by chemical or electrochemical oxidation of aniline performed mainly in the presence of strong mineral acids. The reaction starts with the formation of a cation-radical, which is then coupled with initial aniline molecule with formation of the imide bond. Electrochemical deposition of polyaniline on the electrode is commonly performed by repeating cycling of the potential. In chemical synthesis, (NH_4_)_2_S_2_O_8_ is commonly used. It is reduced to the sulfate anion and does not contaminate the polymer (Cirić-Marjanović, 2013). The following oligomerization involves head-to-tail binding with formation of a linear product existing in various forms, which shape and size depend on the regime of the electrolysis, presence of organic solvents and surfactants and nature of the acid added to the reaction media (Stejskal et al., 2010; Baker et al., 2017). Chemical oxidation can be performed at low temperatures (down to −70°C) to avoid the accumulation of quinoid by-products interfering with electroconductive properties of polyaniline.

The interest to polyaniline is related to its redox properties that depend on the pH and redox potential of the microenvironment. The reactions of intermolecular electron and hydrogen ion exchange (1) are supported by transfer of counter ions that balance the charge of the polymer and also change its intrinsic volume and permeability (Trivedi, 1999). The semi-oxidized form of polyaniline, emeraldine salt, exerts electroconductivity similar to that of doped semiconductors. This property can be used for electric wiring of the enzymes and nucleic acids in biosensor assembly (Dhand et al., 2011). Besides, changes in conductivity caused by the pH shift are recorded in resistive and optical sensors for the measurement of ammonia and weak organic acids (Song and Choi, 2013; Fratoddi et al., 2015). Pure polyaniline exists in emeraldine form only in rather acidic media (pH < 3). This region can be extended by introduction of strong insoluble organic acids in the polymer structure (Karyakin et al., 1999; Lukachova et al., 2003) or by the synthesis of polyelectrolyte complexes with sodium dodecylsulfonate (Andre et al., 2017), polystyrenesulfonate (Luo et al., 2013), carbon nanotubes (Kumar et al., 2018) or even DNA (Shamagsumova et al., 2015) as polyanionic counter ions.

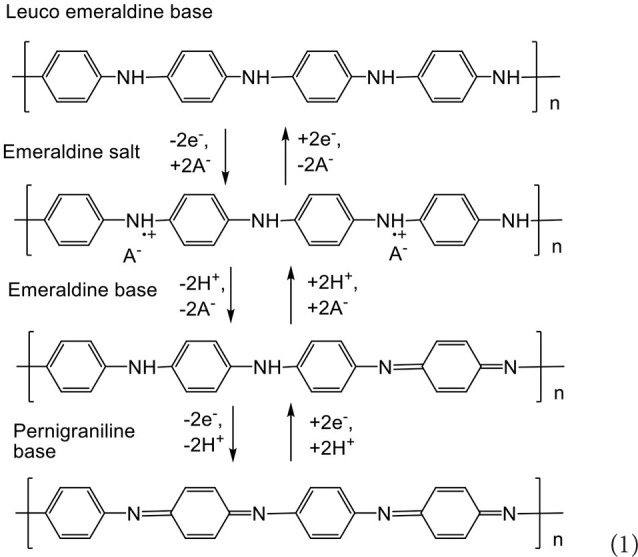



The use of polyaniline in solid-contact potentiometric sensors makes it possible to avoid drawbacks typical for such devices, i.e., slow rate of electron exchange and irreversible response toward alternating analyte concentrations (Lewenstam et al., 1994). Such materials with ion-to-electron conductivity substitute internal filling of conventional ISEs. Solid-contact potentiometric ISEs are easier in manufacture and operation than the commercial ISEs with internal filling and polymer membrane containing ionophore. They do not require long conditioning in an analyte solution prior to use and do not loose workability due to leaching of internal standard solution. Regarding polyaniline, its application in the assembly of solid-contact sensors is limited by necessity in the transfer of doping anions as a part of the redox conversion. This challenge is solved by introduction of acidic groups in the side chains of the polymer (self-doped polyaniline; Jaymand, 2013) or by the addition of high-molecular anionic substances (Zhang et al., 2008). The reversibility of the polyaniline potential can be improved by implementation of conductive materials, i.e., carbon nanotubes (Bavastrello et al., 2004), graphene (Jaworska et al., 2012; Boeva et al., 2014), or noble metal nanoparticles (Shiigi et al., 2012; Lorestani et al., 2015).

Potentiometric sensors based on polyaniline were first applied for the pH measurements. In acidic media, the response is attributed to the shift of equilibrium between emeraldine and leuco-emeraldine forms (see Scheme 1) whereas in basic media the potential is sensitive to the protonation of imine groups that does not result in changes of conductivity and redox reactions (“charge counting” mechanism of the response). In accordance with these mechanisms of potentiometric response, an intermediate pH region exists with reduced influence of the acidity on the polyaniline potential. The sensitivity of the pH measurement can be increased by addition of camphorsulfonic acid (Karyakin et al., 1999). It changes the stoichiometry of the redox reaction normally corresponded to the transfer of two electrons and two hydrogen ions per monomer unit. The over-Nernstian slope of calibration curve is observed for such pH sensors.

The polyaniline doping is a source of the potentiometric activity toward certain anions that are specifically accumulated in the polymer net. Contrary to pH measurements, the reaction with anions is less specific (Hao et al., 2010). In some cases, the addition of analytes in the polymerization mixture improves the performance of appropriate sensors. In other cases, polyaniline is covered with plasticized membrane containing anion-sensitive ionophore, preferably tetraalkylammonium salts (Shishkanova et al., 2005). The determination of nitrate (Khripoun et al., 2006), sulfate (Xing et al., 2017), salicylate (Shishkanova et al., 2014), and sulfonate anionic detergents (Karami and Mousavi, 2004) has been described with polyaniline based potentiometric sensors.

## Thiacalixarenes and pillararenes as ionophores

Thiacalixarenes have found application in the ISEs predominantly for the determination on transient metal cations which are either incorporated in the pseudo-cavity formed by the substituents at the lower rim of the macrocycle or coordinated near the bridging atoms of sulfur (Yushkova and Stoikov, 2009). The inclusion of the metals to form the host-guest complex changes also the aggregation of the macrocycle via distortion of the hydrogen bonds and influences the total charge of the complex in comparison with neutral ionophore molecule. Though the factors mentioned were mostly investigated in organic solvents, they can also affect the potential of appropriate ISEs. Direct application of thiacalixarenes in potentiometric measurement of various metals has been described in rather limited number of articles. Thus, non-substituted thiacalix[4]arene and its derivative with *p-tert*-butyl groups at the upper rim of the macrocycle were implemented in the PVC membrane or directly deposited from vapors on Au electrode for the determination of Co(II) (Gupta et al., 2008) and Cu(II) cations (Ali et al., 2006), respectively. Here and below, roman numerals are referred to the nominal concentration of all the forms of appropriate metals including hydroxycations etc. present in aqueous solutions whereas the plus sign corresponds to a single stable form of the ion (Na^+^, Ag^+^, etc.). It is interesting to note that rather high potentiometric selectivity of both sensors was announced. Mono- and bis(crown-6) thiacalix[4]arenes were successfully used for the determination of Cs^+^ ions in the presence of a wide range of *s*-, *p*-, and *d*-metal cations (Bereczki et al., 2006). The appropriate selectivity (*pK*_*Cs*/*Me*_) varied from −1.2 for Rb to −5.9 for Cu. In this case, the response is pre-determined by geometric conformity of the cation radius and the size of the oxyethylene cycle of the crown-6 binding site of the receptor. The *p-tert*-butylthiacalix[4]arene with two triazole cycles at the lower rim exerted selectivity toward Ag^+^ ions (Omran et al., 2016). The ionophore was implemented in the PVC membrane together with a plasticizer and Na tetraphenylborate. The ISE with internal solution of 0.01 M AgNO_3_ showed linear response to Ag^+^ ions between 7 μM and 8.0 mM [limit of detection (LOD) 3.9 μM].

Comprehensive consideration of selectivity of the solid-contact sensors with thiacalix[4]arene ionophores deposited onto the polyaniline film has been performed in the work of (Evtugyn et al., 2008). Tetrasubstituted thiacalix[4]arenes were synthesized from tetraacid derivatives and contained 2-pyridilamido, morpholide, pyrrolidide, and hydrazide groups responsible for cation binding. Biased potentiometric selectivity coefficients indicated that the binding properties of ionophores depended not only on the nature of the functional groups of substituents but also on the configuration of the macrocyclic core present in *cone, partial cone* and *1,3-alternate* configurations. The lowest LODs of 0.1 μM−20 nM were found for *1,3-alternate* among other isomers and for pyrrolidide derivative among other substituted macrocycles. Assembling of the ion-selective layer was performed by casting the thiacalix[4]arene solution onto the polyaniline. ISEs showed rather high reproducibility of the signal (R.S.D. about 2%) and reversible response toward alternating concentrations of the analyte. The maximum shift of the potential was found for the Ag^+^ ions both in neutral and acidic media. Regarding other metals, the higher pH improved selectivity due to partial hydrolysis of interferences. Only Fe(III) and Hg(II) ions interfered with the Ag^+^ detection. However, their influence could be easily excluded by addition of fluoride and chloride ions, respectively.

**Figure 1:**
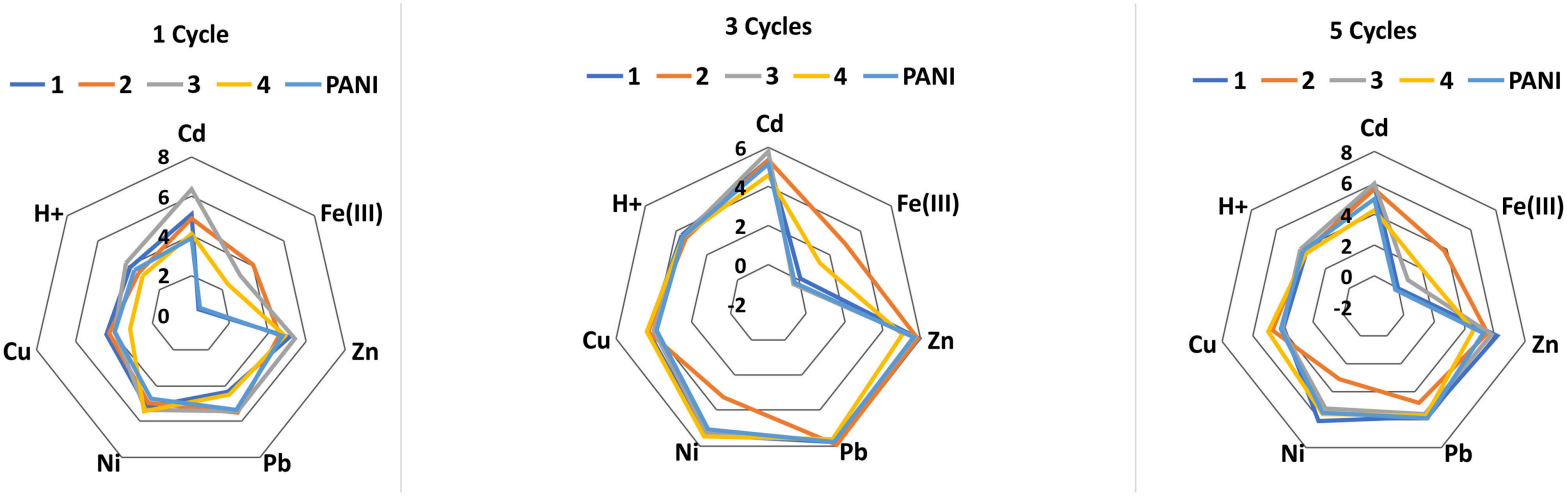
The dependence of the potentiometric selectivity of the solid-contact ISEs with tetrasubstituted *p-tert*-butylthiacalix[4]arenes (10 nanomol per electrode) on the thickness of the polyaniline (PANI) layer expressed by the number of cycles of the potential on the stage of aniline electropolymerization. Color lines correspond to various derivatives of the macrocycle in the assembly of the ISEs: 1-2-pyridilamido (*1.3-alternate*), 2-benzylamide (*cone*), 3-morpholido (*1,3 alternate*), 4-hydrazide (*1,3-alternate*) PANI-no ionophore. The figures near the polygons correspond to -*pK*_*Ag*/*Me*_ values determined by independent solutions method. All the measurements were performed in 0.1 M Na_2_SO_4_ containing 1.0 mM of the metal nitrates.

Deposition of thiacalixarene ionophores partially suppressed the pH sensitivity of the response which is commonly considered as a weak point of the ISEs based on polyaniline. For the same reason, the potentiometric selectivity of the sensors changed with the quantities of the polyaniline. Figure 1 represents changes in the potentiometric selectivity of five ISEs with different assembly of the surface layer toward various metal cations.

The thickness of the polyaniline layer is expressed by the number of potential cycles used on the step of electropolymerization. Both the *pK*_*Ag*/*Me*_-values and the influence of thiacalix[4]arenes were sensitive to the polyaniline thickness. It is interesting to note that even one cycle of the potential in acidic aniline solution was found to be sufficient for deposition of minor amounts of the oligomers providing redox reversibility of the potential and electron-to-ion conductivity. Certainly, such sensors were less stable toward external factors than those with thicker polyaniline film and showed rather a short working period within a day.

No influence of alkaline and alkaline-earth metals was found for such ISEs with no respect of the pH-value and polyaniline film thickness. The differentiation of the response by changing ionophore and measurement conditions was later used for assembling the ET system applied for the discrimination of the foodstuffs reach with oxidizable organic species (see below).

Contrary to the thiacalix[4]arenes, analogous pillar[5]arenes have found limited application in the assembly of potentiometric sensors. This might be due to lower differentiation of the signal toward metal cations involved in supramolecular interactions with this receptor. Thus, pillar[5]arenes with amide and carboxylic groups can bind Li^+^, Na^+^, K^+^, and Cs^+^ with similar association constants determined by UV-viz spectroscopy (Yakimova et al., 2017). The binding efficiency was increased by introduction of the glycylglycide, additional amide, and carboxylic fragments in the macrocycle moiety. Similar results were reported for pillar[5]arenes bearing morpholide and pyrrolidide groups (Shurpik et al., 2014). The association constants of 100–1,000 M^−1^ were determined by electron spectroscopy for alkali metals bonded in 1:1 ratio with insignificant preference of the Li^+^ binding.

The only potentiometric sensor with pillar[5]arene has been described for sensitive determination of Cu^2+^ ions (Stoikova et al., 2015). The solid-contact ISE was prepared similarly to that described for functionalized thiacalix[4]arenes (Evtugyn et al., 2007). The screen-printed carbon electrode was first modified with polyaniline by repeated cycling the potential in the solution of the monomer in concentrated sulfuric acid. After that, freshly prepared solution of unsubstituted pillar[5]arene was dropped onto the working electrode and left to dry at ambient temperature. The electrode made it possible to determine from 1 μM to 10 mM of Cu^2+^ ions (LOD 0.3 μM). The ISE was successfully applied for detection of Cu(II) ions in the polyvitamin drops “Complivit” and Bordeaux mixture used as a fungicide in vineyards, fruit farms and gardens. Interfering effect of the Fe(III) and Ag^+^ ions was referred to the redox reaction between the polyaniline and metal ions determined. In case of silver, the formation of metal nanoparticles in the reaction with polyaniline was also confirmed by transmission electron microscopy (Smolko et al., 2014).

## Solid-contact potentiometric sensors in binary mixtures

Substituted thiacalix[4]arenes meet the requirement of cross-selectivity in multicomponent media required for assembling the potentiometric ET systems. This was first shown on the example of simultaneous determination of two cations [Ag^+^ – Hg(II) and Ag^+^ – Fe(III)] in binary mixtures. Both Hg(II) and Fe(III) ions interfere with silver and could not be determined by a single solid-contact sensor (Evtugyn et al., 2007). The ET consisted of nine individual electrodes manufactured on the same platform of screen-printed carbon electrodes covered with electropolymerized polyaniline. Three electrodes were covered with the pyridine derivative **1** in *1,3-alternate* conformation and three others with structurally similar derivative **2** in *cone* conformation (2).

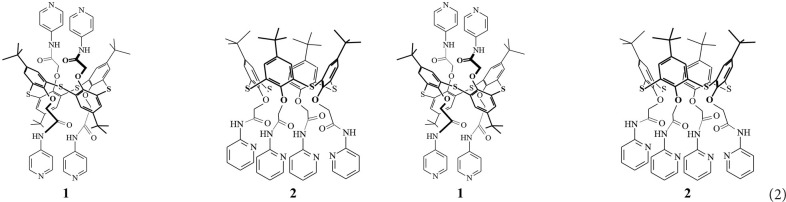



Last three electrodes were used as they were without additional deposition of ionophores to take into account the shift of the potential attributed to the polyaniline. The signals of the ISEs described were recorded in a set of model binary solutions of different concentrations without intermediate averaging.

The ET described was first explored to predict the signals of individual sensors at a certain pH by modeling the response using linear and on-linear models created on a known set of binary standard solutions (Evtugyn and Stoikov, 2016). For Ag^+^ – Hg(II) mixture, satisfactory results have been obtained with non-linear model (3) followed from the Nikolsky-Eisenman equation and describing mutual influence of the cations in binary mixture on the ISE potential.

(3) $$y_{i}=a_{i}^{o}+b_{i}\ln(k_{i}c_{1}+c_{2}+k_{i,12}c_{1}c_{2})$$

Here *c*_1_ and *c*_2_ are the concentrations of the cations, *i* is the number of the ISE and *a*_*i*_, *b*_*i*_, and *k*_*i*_ are the parameters to be extracted from the experimental data by minimization of the mean-square error function. Measurements were performed in weakly acidic media (pH = 1÷5) corresponding to the real assay conditions. The pH range covers transient area corresponded to the reversible transfer of the polyaniline between the conductive emeraldine and non-conductive oxidized form. For this reason, sensor potential can be sensitive to minor pH changes and appropriate shifts of hydrolytic equilibria of Fe(III) and Hg(II) species. Complex pH effect was ascribed by the pH sensitivity of the above parameters *a*_*i*_, *b*_*i*_, and *k*_*i*_ (*i* = 1÷3 for three types of ISEs) (4).

(4) $$\begin{array}{l} a_{i}^{0}(pH)=a_{10}+a_{11}\cdot pH+a_{12}\cdot pH^{2}\\ b_{i}(pH)=a_{20}+a_{21}\cdot pH+a_{22}\cdot pH^{2}\\ k_{i}(pH)=a_{30}+a_{31}\cdot pH+a_{32}\;\cdot pH^{2} \end{array}$$

The partial coefficients *a*_*ij*_ were fitted by minimization of the mean-square error function (5).

(5) $$F^{\left(i\right)}(a_{i}^{o}(pH),\;b_{i}(pH),\;k_{i}(pH))={\sum_{j}\left(\frac{y_{j}^{\left(i\right)}-{\hat{y}}_{j}^{\left(i\right)}}{y_{j}^{\left(i\right)}}\right)}^{2}$$

Direct additive model has shown good prediction of individual responses. After that, the concentrations of Ag^+^ and Hg(II) present in binary solutions were estimated within the range from 10 mM to 10 μM by a number of statistical methods including generalized additive model (GAM), partial least square (PLS) analysis, and artificial neural network (multilayer perceptron, MLP). The root-mean square error did not exceed 5.6% of logarithm of concentration of each ion. Best results were obtained with MPL consisted of two hidden layers with five and three neurons, respectively.

Similar consideration of Ag^+^-Fe(III) binary solutions resulted in better prediction of the cation concentrations against Ag^+^-Hg(II) mixture due to the bigger difference in the shape of the calibration curves obtained separately for each metal cation. The signal toward Fe(III) appears due to reversible redox conversion of the polyaniline and results in the S-shaped calibration curve with a rather narrow linear piece in the middle of the concentration interval.

The prediction of metal concentrations with GAM was based on the Equation (6).

(6) ln C=Const+s(1)+s(2)+s(3)+ε,

where s(1), s(2), and s(3) are smooth functions describing the dependence of the individual sensor signal on the logarithm of the cation concentration, and ε is the random error with zero mean value. The GAM retains additive response summation approach but takes into account non-linear behavior of each sensor toward a certain analyte. The prediction model provided 90.6% of explained deviance for Fe(III) concentration and 70.3% for Ag^+^ ions. The MLP model showed better quality of prediction than linear model especially for Fe(III) concentration. The PLS regression showed that the prediction is mostly based on the signal of the sensor with the macrocycle **1** in *1,3 alternate* configuration whereas the polyaniline with no ionophore takes into account the hydrolytic instability of the cations.

## Multisensor systems for food assessment: concept description

Solid-contact ISEs with the thiacalix[4]arene derivatives showed variable response toward many transient metal cations that depended on many factors including acidity and buffering properties of the media (Evtugyn et al., 2007, 2008). Additive model of the ISE response showed satisfactory agreement with the experimental data. However, detectable concentrations of most heavy metals were rather high for direct application of such sensors in environmental monitoring. Meanwhile, redox response of the Fe(III) ions caused by its reaction with polyaniline can be applicable for indirect characterization of many samples containing readily oxidized compounds that could affect the signal. This offers new opportunities for the use of such electrodes in the quality assessment of foodstuffs, fruits, vegetables and beverages. Indeed, ferric reducing activity measurement is a conventional parameter applied for spectrophotometric determination of “antioxidant power” (Benzie and Strain, 1996). Electrochemical analogs include ferricyanide based protocols with voltammetric signal measurements (Berker et al., 2010; Klõšeiko, 2016).

In case of solid-contact ISEs on polyaniline platform, the detection of readily oxidized species is simplified by non-linear shape of the Fe(III) calibration curve. It can be expected, that together with the antioxidants, partial hydrolysis of the Fe(III) species and their complexation with organic substances present in organic matter would alter the potential of the sensors/ This offers possibilities for extraction of new information on the sample content, origination, nutritive value etc.

The possibility to monitor the antioxidants with the solid-contact ISEs based on polyaniline and thiacalix[4]arene ionophores in the presence of Fe(III) ions was confirmed by the analysis of some typical antioxidants like ascorbic, malic and oxalic acids, quercetin, and hydroquinone (Evtugyn et al., 2010). Their LODs varied from 2 μM (quercetin) to 0.1 mM (oxalic acid). Thiacalix[4]arenes **2** and **3** (7) affect the sensitivity of the antioxidant determination. More lipophilic derivative **3** with long alkyl radicals in the substituents at the lower rim decreased the LODs of neutral analytes by 2–4 times against those of ascorbic, malic and oxalic acids. Meanwhile pyridine derivative **2** showed similar LOD values for all the antioxidant tested.

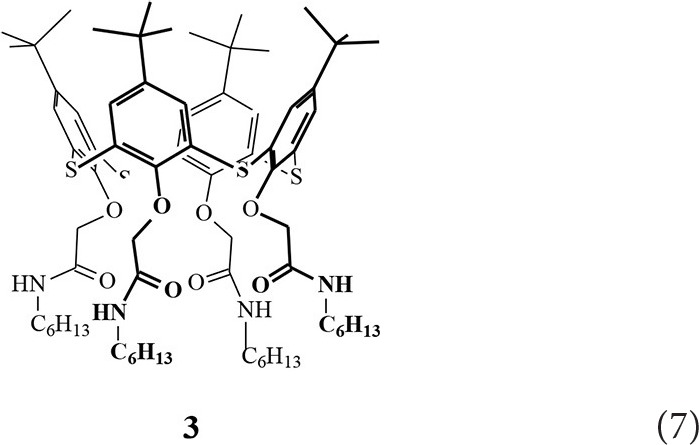



**Figure 2:**
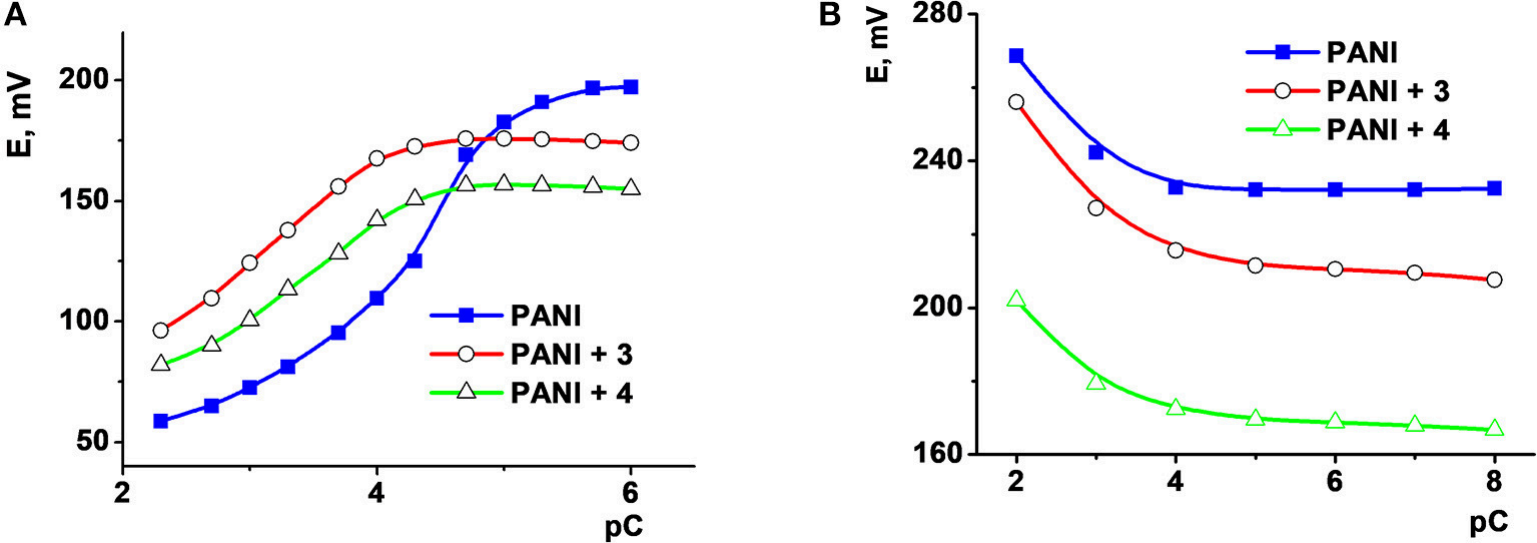
The dependence of the potential of ISEs based on polyaniline (PANI) and thiacalixarenes **2** and **3** on the concentration of ascorbic acid **(A)** in apple juice diluted in 1:20 v/v ratio with 0.1 M Na_2_SO_4_ and that of citric acid **(B)** in 0.1 M Na_2_SO_4_ solution. Adapted from (Evtugyn et al., 2010), with permission of Elsevier.

Different sensors respond to organic species present in the samples in a different manner. Figure 2A shows the signals recorded in the apple juice spiked with different amounts of ascorbic acid and a constant Fe(III) concentration. Moreover, citrate ion shifts the potential of the sensor in the opposite direction (Figure 2B) because of the formation of negatively charged complex with Fe(III) ions and its electrostatic accumulation onto the polyaniline surface.

**Figure 3:**
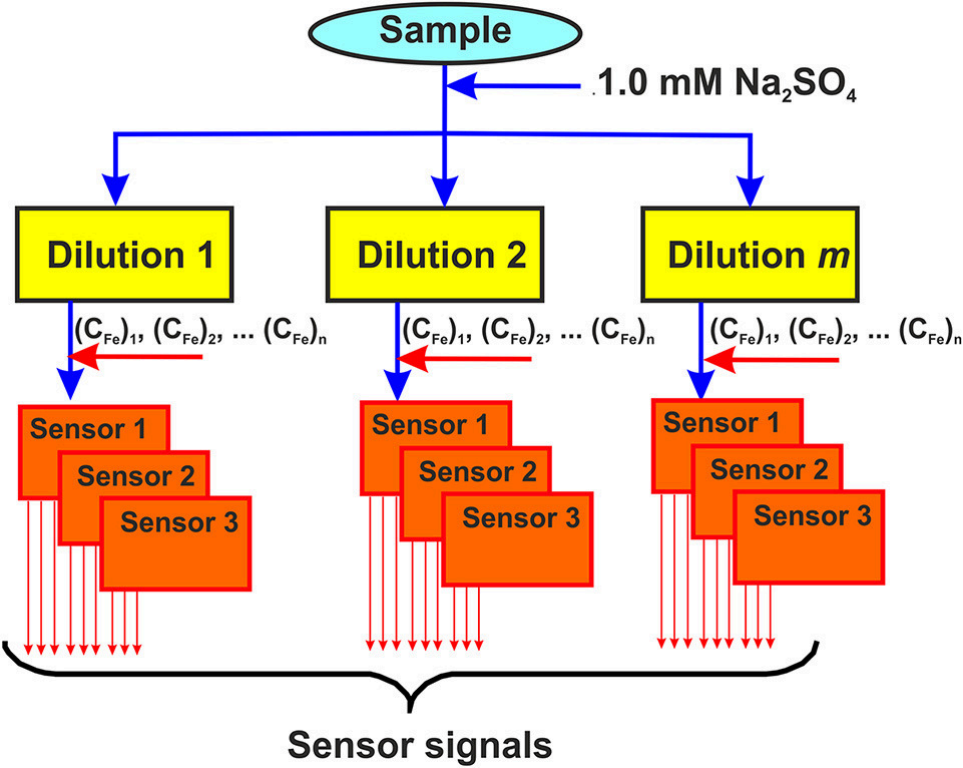
Principal scheme of the potentiometric ET application. Solid-contact ISEs are based on the polyaniline and thiacalix[4]arene ionophores.

Variety of the ISEs responses to various components of real samples, e.g., fruits, vegetables, foodstuffs, beverages etc., made it possible to propose the following protocol for the assessment of the above products and their discrimination in accordance with *a priori* and *a posteriori* formulated specifiers (Figure 3):

- The sample is diluted in a certain ratio with supporting electrolyte to stabilize its ionic strength and reach antioxidant concentration required;
- The aliquot of the Fe(III) salt is added into the diluted sample to reach a constant concentration in all the dilutions;
- The solid-contact ISEs based on the polyaniline and the thiacalix[4]arene ionophores are inserted in the diluted solutions and their potentials are measured against the same reference electrode (Ag/AgCl);
- A series of such measurements is performed with a certain number of sample dilutions and Fe(III) concentrations.

The number of measurements, the number of ISEs in the ET system and the choice of ionophores are the subject of investigations conducted with standard solutions (known samples). In most cases, six or nine ISEs with two or three ionophores are combined in the ET system and one or two different concentrations of Fe(III) ion are added prior to potential measurement. The signals of all the individual sensors with the same thiacalix[4]arene derivative were then processed without intermediate averaging to take into account their variability. The dilutions of the samples commonly varied from 1:1 to 1:200. The examples of the application of the ET described are given below.

## Fruit juices

Fruits and fruit juices are most important natural sources of antioxidants, vitamins, and other nutrients demanded for the human health. Meanwhile the assessment of the fruits based on the potentiometric ETs has been described only in few works. Thus, the discrimination of apple variables by the content of organic acids and sugars was demonstrated with the ET comprising of 16 potentiometric sensors in combination with Fourier transform infrared spectroscopy with attenuated total reflection (ATR-FTIR) and HPLC data (Rudnitskaya et al., 2006). The classification of the apple varieties was performed using principal component (PCA) and discriminant analysis. The calibration of organic acids and sugars was performed with PLSs regression. Peach, orange, pineapple and grapefruit juices were classified with potentiometric multisensor system consisted of copper, tin, iron, aluminum, brass, and stainless-steel wires (Lvova et al., 2006a). The classification confirmed by PCA was based on different content of organic acids. Thirty-six potentiometric sensors with PVC membranes have been employed for classification of 16 commercial fruit juices and fruit containing non-alcoholic drinks (Dias et al., 2011). The quantitative determination of fruit content in the beverages was performed using multiple linear regression and PLS.

**Figure 4:**
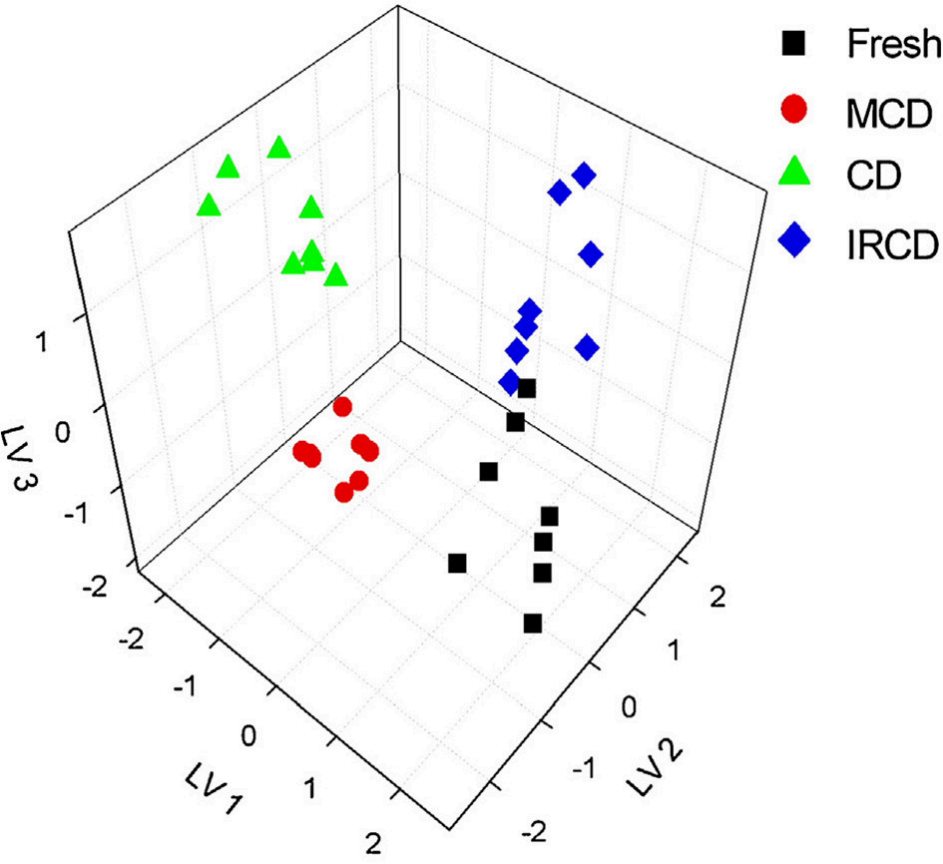
The 3D PLS-DA score for classification of fresh and dried apples with potentiometric ET. LV—latent variables, colored symbols correspond to various drying regimes. Kutyła-Olesiuk et al. (2013) with permission of Elsevier.

Extracts from dried apples were tested using ET consisting of Ca^2+^, Na^+^, K^+^, cation- and anion-selective ISEs, Au voltammetric, and conductometric sensors (Kutyła-Olesiuk et al., 2013). The efficiency of qualitative and quantitative analysis was estimated by root mean squared error (RMSE) and parameters of correlation between real and PLS predicted samples. The 3D score plot obtained by PLS-DA for discrimination of fresh apples and dried ones is shown in Figure 4 for potentiometric ET. The recognition ability was estimated as percentage of correct prediction of the apple sample tested. The results obtained with potentiometric and hybrid ET showed similar sensitivity but the classification was reliable with the hybrid ET system.

**Figure 5:**
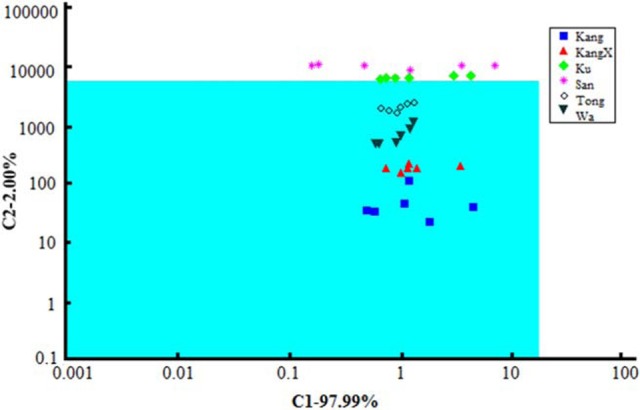
SIMCA plot of ASTREE sensor signals of different brands on orange beverages (Liu et al., 2012), with permission of Wiley Periodicals, Inc.

Commercial sensor array based on seven field-effect transistors (FETs) with cross-selective membranes (α-ASTREE, Alpha-M.O.S.) was successfully used for discrimination of beverages containing orange juice (Liu et al., 2012). The signals of the ET system were correlated with the results of reference methods. The significant parameters specifying brand of the drink were first determined by single-factor analysis of variance (ANOVA). Among them, amino acid nitrogen, reducing and non-reducing sugars, sugar-to-acid ratio, total acid, total solids, and total soluble sugar were considered. Six brands were classified by ET signal processing using a number of supervised and unsupervised methods [PCA, canonical discriminant analysis, cluster analysis, and soft independent modeling of class analogy (SIMCA)]. As an example, SIMCA plot of seven sensor signals related to different brands is shown in Figure 5. One could see, four brands (Kang, KangX, Tong, and Wa) located in the green quadrate region demonstrate high similarity. This corresponds to the PCA score that indicated two groups of similar brands classified in accordance with their physicochemical parameters.

e-Nose and ET systems were combined for discrimination of a wide number of Tunisian fruit juices (46 brands available in local market) (Haddi et al., 2014). e-Nose consisted of five commercially available MOS gas sensors and the ET of six potentiometric electrodes. The PCA showed that taken separately e-Nose and ET did not provide identification of each juice. Meanwhile its combination and the use of fuzzy ARTMAP neural nets resulted in satisfactory discrimination among samples tested except two juices.

**Figure 6:**
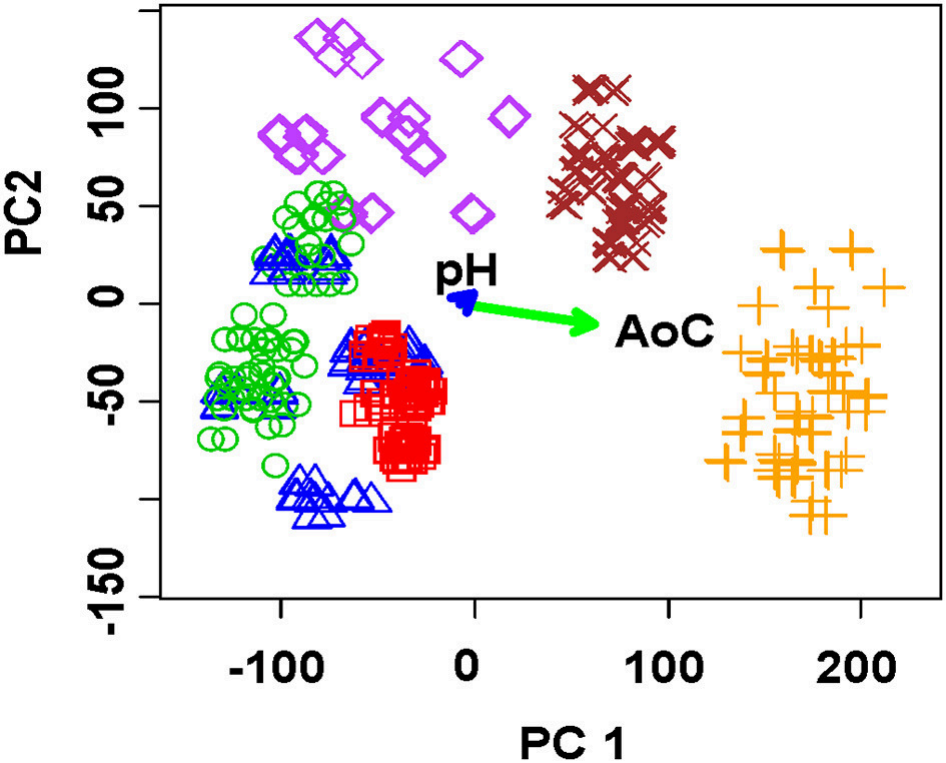
PCA scores plot (two first principal components) of apple juice brands obtained with the ET system comprising of nine solid-contact ISEs on the base of the polyaniline and thiacalix[4]arene ionophores. AoC—antioxidant capacity. Two sample dilutions (1:70 and 1:100) and one Fe(III) concentration (1.0 mM) (Evtugyn et al., 2010), with permission of Elsevier.

In case of solid-contact ISEs based on polyaniline and thiacalix[4]arene derivatives, similar classification can be achieved by much lower number of sensors. Thus, the discrimination of six commercial apple juices was performed using nine sensors, three of which contained two thiacalix[4]arenes **2** and **3** and last three sensors only polyaniline (Evtugyn et al., 2010). The juices taken from 200 mL packages were diluted in 1:70 and 1:100 ratio and spiked with 0.1 and 1.0 mM Fe(NO_3_)_3_. Thus, the set of independent variables comprised of 2 × 2 × 9 = 36 points (signals of individual ISEs) for each juice tested. The classification in accordance with the brand name was performed using PCA. The explained variance by two first principal components was found to be 97.5% (78.8% for one Fe(III) concentration and two dilutions of the juices, Figure 6).

Simultaneous determination of total antioxidant capacity confirmed the hypothesis about predominant influence of antioxidants on the sensor signals. The difference in the response toward certain juices can be referred to the minerals and vitamins added to natural fruits by producers and/or to rather small portion of natural juice in beverage tested (25%). Contrary to that, pH of the samples did not significantly affect the discrimination, probably due to rather narrow range of its variation (pH 3–4). The possibility of discrimination of apple juices and their brand name prediction were considered by linear discriminant analysis (LDA) showed 73–100% of true predictions depending on the initial learning sets.

The same approach was used for the discrimination of extended set of fruit juices (apple, lemon, grapefruit, grape, and orange) and fruit containing beverages (35 samples in total) that involved commercial packages and freshly pressed juices. Several samples pressed from fruits were then stored for different time (1–4 days) to estimate changes in their quality. The PCA showed satisfactory separation of all the samples by testing two juice dilutions with two different Fe (III) concentrations. Separation of samples by the ET signals was estimated with χ^2^ statistics. As an example, Table 1 represents comparison of various freshly pressed juices with different storage period. Black cells of the table correspond to full separation of appropriate juices, gray cells to their partial separation and white cells to fully concurrent classes on PCA scores.

**Table 1:** χ^2^ statistics of freshly pressed juices (95% significance level, critical value for three sensors is 7.8 assuming normal data distribution).

**Juice—storage period (days)**	**1**	**2**	**3**	**4**	**5**	**6**	**7**	**8**	**9**	**10**	**11**	**12**	**13**	**14**	**15**	**16**	**17**	**18**	**19**	**20**	**21**
1. Orange-0	0.0																				
2. Orange-1		0.0																			
3. Orange-2			0.0																		
4. Grape-0				0.0																	
5. Grape-1					0.0																
6. Grape-2						0.0															
7. Grape-3							0.0														
8. Grape-4								0.0													
9. Grapefruit-0									0.0												
10. Grapefruit-1										0.0											
11. Grapefruit-2											0.0										
12. Grapefruit−3												0.0									
13. Green apple-0													0.0								
14. Green apple-1														0.0							
15 Green apple-3															0.0						
16. Green apple-4																0.0					
17. Lemon-0																	0.0				
18. Lemon-1																		0.0			
19. Red apple-1																			0.0		
20. Red apple-2																				0.0	
21. Red apple-3																					0.0

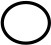
− 0 < . χ^2^ < 5; 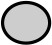
− 5 < . χ^2^ < 10; 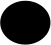
− χ^2^ >10

Most of the samples are fully separated, the concurrent samples belong to congenial fruits (orange and grapefruit). It is interesting to note that the χ^2^ statistics indicated different stability of the juices during the storage period. Only grapefruit and lemon kept separation of their ET signals during more than 1 day storage. This might be related to rather high acidity of these samples against other juices tested. Reduced number of parameters, i.e., application of one dilution or of one Fe(III) concentration, can be applicable for discrimination of a smaller number of samples based on simpler rules, e.g., separation of two known brands of commercial grape juices or separation of 100% fruits juices from fruit containing beverages.

## Tea infusions

Tea is reach with antioxidants which availability and influence on taste depend on fermentation technology, tea origination, and extraction conditions. Potentiometric ETs based on PVC and polyurethane membranes were applied to distinguish black and green tea and coffee (Lvova et al., 2003). Similar plasticized membranes have been employed in the assembly of cation- and anion-selective ISEs combined together with commercial pH- and redox-sensitive electrodes in the ET system for differentiation of black and herbal tea based on PCA method (Cuartero et al., 2015).

Twenty-two potentiometric sensors including 10 anion-selective, 8 cation-selective ISEs with PVC membranes, 3 sensors made of chalcogenide glass with redox sensitivity and one standard pH electrode were applied for classification of 34 black tea samples available in Russia and Spain markets (Khaydukova et al., 2015).

Alpha M.O.S. system with six potentiometric semiconductor sensors was used for evaluation of taste sensory attributes (heavy flavor, thick flavor, sweet flavor, fresh flavor, mellow flavor, astringency, tenderness, stale flavor, purity, and fired flavor) against sensory profile test of 8 samples of black and 8 samples of green tea from different China provinces (He et al., 2009).

**Figure 7:**
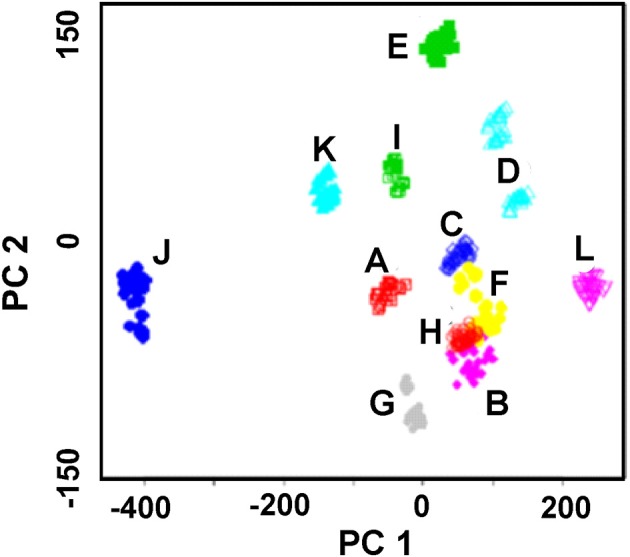
PCA scores plot (two first principal components) of green tea infusions obtained with the ET system comprising of nine solid-contact ISEs on the base of the polyaniline and thiacalix[4]arene ionophores. Sample dilutions 1:1 and 1:20, Fe(III) concentration 0.1 and 1.0 mM. Reproduced from Evtugyn et al. (2011a) with permission of J.Wiley & Sons.

The use of solid-contact ISEs based on polyaniline and thiacalix[4]arene ionophores provides discrimination of green tea infusions with a small number of samples/measurements (Evtugyn et al., 2011b). The multisensor system comprised of nine screen-printed electrodes covered with polyaniline and two thiacalix[4]arenes **2** and **3**. The dilution optimal for discrimination was much smaller than that of fruit juices described above because of much lower content of antioxidants and complexing agents in the rea infusions. The sample set involved eleven natural and flavored green tea brands and one of oolong tea, which antioxidant capacity determined by coulometry varied from 0.45 to 5.89 Q/100 mL. Figure 7 shows PCA score plot obtained with two dilutions and two Fe(III) concentrations. The explained variance differed from 80 to 97% for various initial data sets. No significant influence of antioxidant capacity on discrimination results was fund. Probably, complexation of Fe(III) ions and direct effect of polyphenols on the polyaniline could mask such a dependency.

The LDA confirmed high efficiency of discrimination resulted in 100% prediction of the green tea brand both for the full data set (two dilutions, two concentrations of Fe(III) ions and nine sensors, 36 signals per one sample) and their reduced replicas (one dilution and one Fe(III) concentration, nine signals per one tea infusion).

## Alcoholic drinks

Wine and beer are most frequently tested by potentiometric ETs based on various principles of signal generation (Zeravik et al., 2009). Many components influencing odor, taste, flavor and other properties directly affecting the cost of wine can alter the signals of individual sensors and hence provide discrimination of wines in accordance with qualitative and quantitative criteria. Thus, 20 samples of Barbera d'Asti and in 36 samples of Gutturnio wine were discriminated in accordance with their denomination and vintage by the ET of 23 potentiometric sensors (Legin et al., 2003). Simultaneously, quantitative parameters, e.g., total and volatile acidity, total polyphenols, glycerol, pH, ethanol, and tartaric acid concentrations were determined using multivariate calibration. Similar research has been performed with white wines using solid-contact sensors with PVC membranes containing metalloporphyrins (Verelli et al., 2007). The ET was used for classification of wines in accordance with their origin and content of some components, e.g., malic and lactic acids, SO_2_, polylphenols etc. ET assembled from similar sensors were applied for assessment of Madeira (Rudnitskaya et al., 2012) and New Zealand (Kirsanov et al., 2012) wines in accordance with their age (maturation). The capabilities of traditional potentiometric ET systems were extended by comparison of the results with the HPLC analysis (Rudnitskaya et al., 2010), FTIR spectroscopy (Costa et al., 2015) and humid electronic nose (Gil-Sánchez et al., 2011). Most of the ETs described offer opportunities for the wine classification and determination of common organic species like organic acids, polyphenols, other antioxidants etc. Besides, specific parameters of wine quality like bitterness (Rudnitskaya et al., 2010) or astringency (Costa et al., 2015) can be taken into account. The ETs make it possible to monitor technological processes (maceration with oak chips; Rudnitskaya et al., 2017) or wine spoilage (Gil-Sánchez et al., 2011).

Beer is another subject of the ET application mostly directed to the discrimination of certain types (light and dark beer) or brands. The discrimination is performed mostly with ISEs involving plasticized PVC membranes sensitive to inorganic cations (Na^+^/K^+^, Ca^2+^, ${\text{NH}}_{4}^{+}$) and anions (H_2_${\text{PO}}_{4}^{-}$, Cl^−^). Cation- and anion-selective membranes containing lipophilic salt but not ionophore can be added. Contrary to wine testing, the response of such ISEs is not attributed to specific beer components but is indirectly influenced by lipophilic and surface-active species present in the samples. On the one hand, this makes the ET approach universal and applicable to a wide variety of beer sorts. On the other hand, such mechanism assumes quasi-reversible response and sometimes leaves room for artifacts and uncertainty in interpretation of the results.

Solid-contact ISEs with planar Au electrodes covered with the PVC membranes have been used for flow-through assessment of beer originated from different manufacture lots (Ciosek and Wróblewski, 2006). The combination of PLS and artificial neural networks resulted in 83% of true predictions.

**Figure 8:**
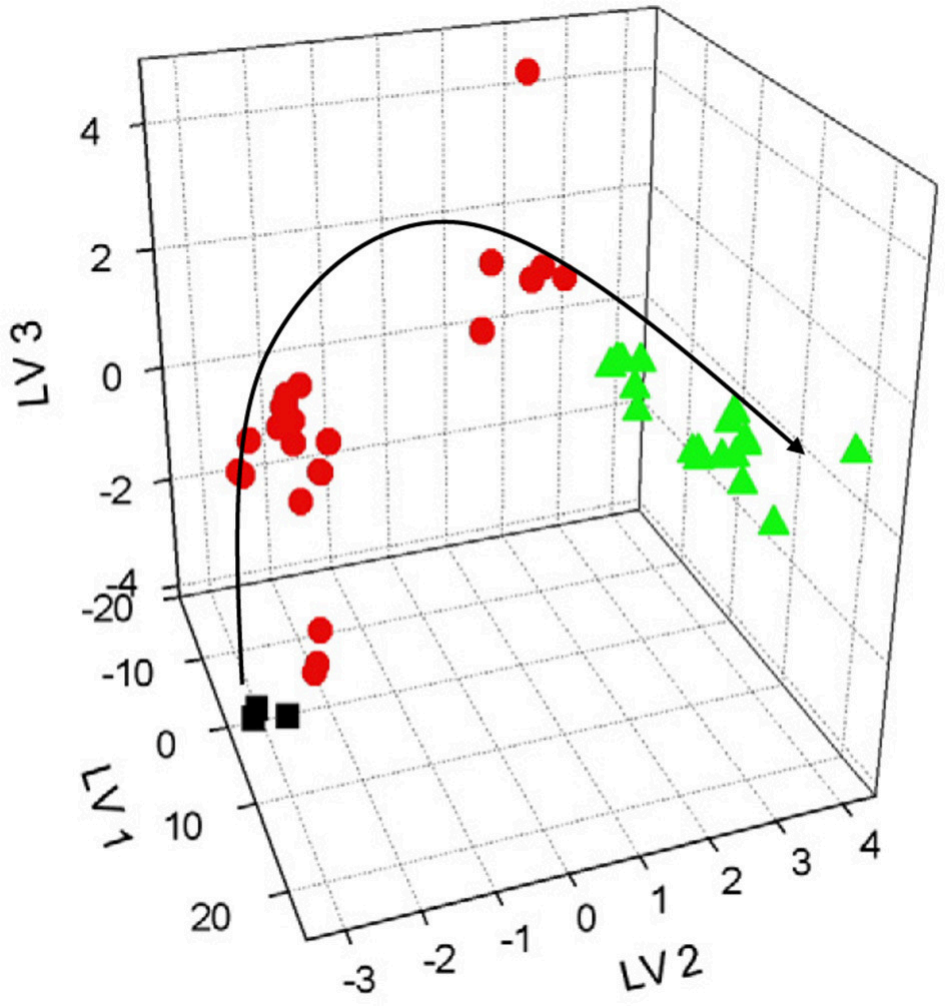
PLS-LA plot of beer samples for hybrid ET data with three latent variables, different colors indicate various fermentation and beer aging samples.

Similar potentiometric ET combined with voltammetric sensor has been applied for characterization of beer fermentation (Kutyła-Olesiuk et al., 2012). The results obtained were processed using PLS and PLS-DA. Both methods allow tracing changes of chemical images linked with overall composition of the fermentation media leading to discrimination of individual samples and estimation of fermentation and maturation time. Figure 8 shows typical chemical image obtained by data processing. Graphs show distinction between samples different in fermentation and aging periods. The discrimination ability was assessed the F factor. It describes the variance between different classes and variances between particular classes. For three latent variables, the variances can be expressed as Euclidean distances in the pattern space (8).

(8) $$F=\frac{\frac{\sum_{i=1}^{k}\sqrt{{\left(x-x_{ji}\right)}^{2}+{\left(y-y_{ji}\right)}^{2}+{\left(z-z_{ji}\right)}^{2}}}{k-1}}{\frac{\sum_{i=1}^{k}\sum_{j=1}^{n_{j}}\sqrt{{\left(x-x_{ji}\right)}^{2}+{\left(y-y_{ji}\right)}^{2}+{\left(z-z_{ji}\right)}^{2}}}{\sum_{i=1}^{k}\left(n_{j}-k\right)}}$$

Here *k* is the number of a class, *i* is the number of sample, *j* is the number of sample in *i*-th class, *n* is the number of samples in *i*-th class, *x, y*, and *z* are sensor response for *j*-th sample in *i*-th class, *x*_*ji*_, *y*_*ji*_, and *z*_*ji*_ are the mean values of sensor response for samples between different classes, *x*_*i*_, *y*_i_, and *z*_*i*_ are mean value variances in particular class. Increasing F factor indicates bigger difference between classes. The hybrid ET showed three-fold decrease of the RMSE value against potentiometric ET.

The ET comprising of 14 ISEs with plasticized PVC and chalcogenide glass membranes was utilized for discrimination of Belgian and Dutch beers (Polshin et al., 2010). Correlation between the signals of individual sensors and several beer parameters (fermentation degree, alcohol content, pH, bitterness, color, polyphenol, and CO_2_ content) was established and bitterness of real beer brands evaluated using PCA and canonic correlation analysis.

Most of the ET described consist of the ISEs with PVC membranes. New approaches to assembling of the multisensors have been appeared in the last years. Thus, planar microelectrodes with silver trackers have been produced from Whatman™ paper and then covered with PVC films containing various ionophores (Neru and Kubota, 2016). The discrimination of wine and bear was performed by measuring Ca/Mg and K/Na amounts together with total cation and anion concentrations and some inorganic anions present in the samples. The electrodes were impregnated in the sample to minimize the volume required for measurement. The classification was performed with PCA and multivariate calibration with principal component regression and K-nearest neighbors (KNN). Besides wine classified by different varieties of grape, the beer samples were tested by their brand and type. The ET proposed makes it possible to estimate pH, alcohol content, carbohydrate, and caramel dye as well as fermentation type of beer.

Less attention has been paid to strong drinks like vodka or eaux-de-vie. Ethanol content was determined with the ISEs based on plasticized membranes with tetraphenylporphyrin complexes of Ni, Co, Mn, and Fe (Lvova et al., 2006b). PCA and SIMCA were applied for the discrimination of alcoholic spirits and their classification. The ET showed sensitivity toward ethanol among other aliphatic alcohols and made it possible to distinguish various beer and wine sorts.

Chalcogenide glass and plasticized anion- and cation-selective membrane electrodes have been specified for the assessment of ethanol and hot drinks (Legin et al., 2005). The ET signals were processed using PCA and PLS regression. The following samples were successfully discriminated: synthetic and alimentary grain ethanol in different degrees of purification, vodka from various Russian regions (12 samples), and cognac (six samples).

**Figure 9:**
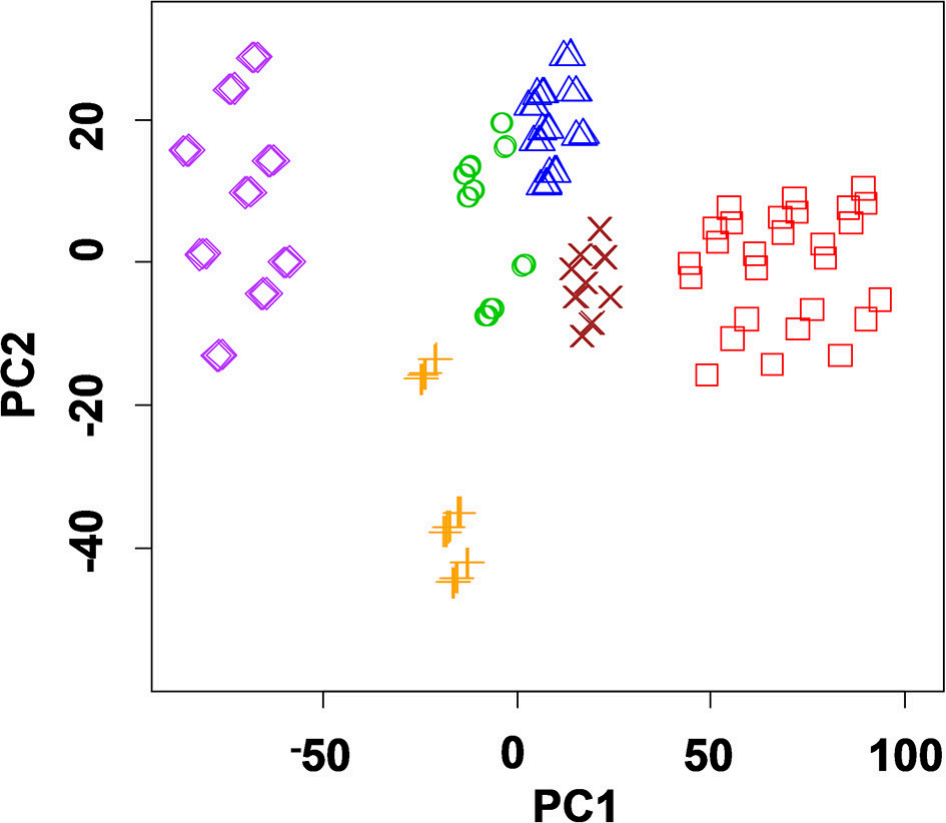
PCA scores plot (two first principal components) of herbal liqueurs obtained with the ET system comprising of nine solid-contact ISEs on the base of the polyaniline and thiacalix[4]arene ionophores **2** and **3**. Sample dilutions 1:10, 1:20, Fe(III) concentration 0.1 mM (Evtugyn et al., 2010), with permission of Elsevier.

In case of solid-contact ISEs with polyaniline and thiacalix[4]arene ionophores, discrimination of alcoholic drinks can be achieved. This was first shown by discrimination of herbal liqueurs containing 28–40% of ethanol and 10–20% of sugar (Evtugyn et al., 2010). Measurements were performed with 1:1 and 1:20 dilution of the samples in the presence of 1.0 and 10 mM Fe(III) ions. Recognition of six liqueurs illustrated by PCA score plot is shown in Figure 9. The LDA provided 82% prediction in case of nine measurements (one dilution with one Fe(III) ion concentration) and 92% for 18 measurements (two dilutions with one Fe(III) ion concentration).

Fifteen brands of beer were classified using ET based on screen-printed electrodes covered with polyaniline and three thiacalix[4]arene receptors with long-chain alkyl- (-C_18_H_37_), amino- and pyrrolidide groups in *1,3-alternate* conformation (Stoikova et al., 2014). The selection of ionophores among macrocycle derivatives was made by ranking individual sensors by prediction efficiency in accordance with the beer brands performed with LDA. Not <70% of successful prediction were obtained with the ionophores tested. In case of the above three thiacalix[4]arenes, the efficiency of the prediction with the ET system reached 100%. Simplified multisensor system with two electrodes allows reliable distinguishing between light and dark beer sorts. In this case, screen-printed carbon electrodes can be used as transducers instead of glassy carbon electrodes.

**Figure 10:**
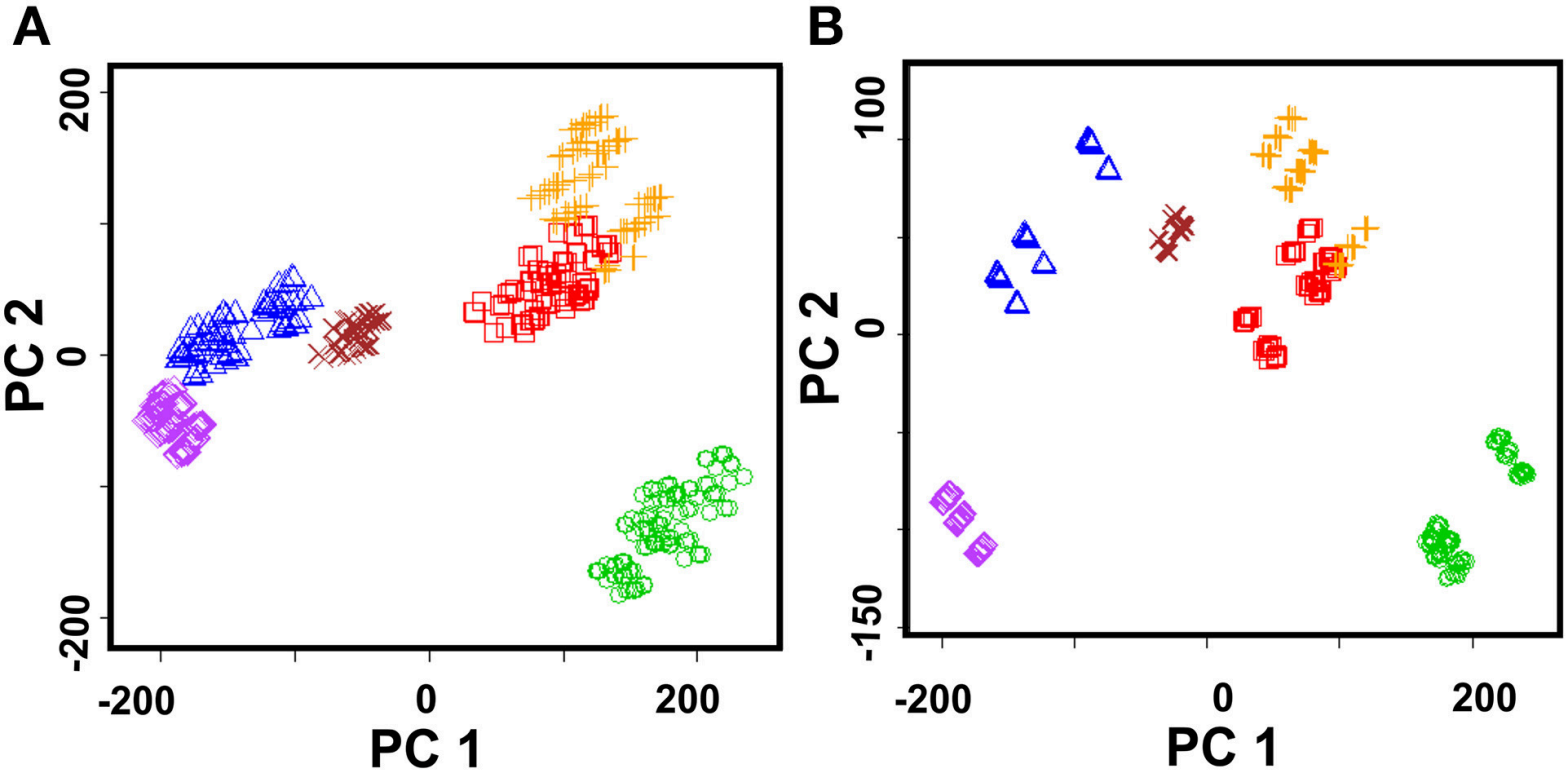
PCA scores plot (two first principal components) of whiskey (Jim Beam, Jack Daniels, Jameson, Chivas Regal, Red Label, Black Label) obtained with the ET system comprising of nine solid-contact ISEs on the base of the polyaniline and thiacalix[4]arene ionophores **2** and **3**. **(A)** Sample dilutions 1:1 and 1:20, 1.0 mM Fe(III), explained variance 84.8%; **(B)** Sample dilution 1:5, 1.0 mM Fe(III), explained variance 92.9%.

Various hot drinks were successfully discriminated using the ET system with solid-contact sensors covered with polyaniline and thiacalix[4]arenes **2** and **3** (Evtugyn et al., 2011a). The ET system was applied for discrimination of four brands of vodka from local Russian and EU distilleries, two brands of cognac, and six brands of whiskey. Besides, 40% grain ethanol of various quality was added as a model of vodka falsifications. The efficiency of discrimination was considered using PCA for different number of sample dilutions. Best results were obtained with two dilutions and one or two concentrations of the Fe(III) ions (18 and 36 points in the data set). The explained variance reaches 87–90%. Increasing number of dilutions to 3–5 decreased the explained variance of the PCA score to 70%. Similar results were obtained for particular hot drinks. As an example, Figure 10 shows the PCA score plot for whiskey samples.

The examples described above do not exhaust the application of potentiometric ET systems for foodstuffs discrimination based on their antioxidant properties. Thus, fermentation processes classified by potentiometric and hybrid ET systems are summarized and discussed in (Ciosek and Wróblewski, 2015). Food analysis with artificial senses based on different principles with particular emphasis to data mining protocols is considered in (Sliwinska et al., 2014). New approaches to the use of biochemical sensors for ET discrimination of complex media and data mining systems commonly used in such works are carefully considered in Cetó et al. (2016).

## Conclusion

Solid-contact ISEs with the macrocyclic ionophores offer broad opportunities for assessment of various foodstuffs on the base of antioxidant content and for their discrimination by brand name or origination. Such ET applications coincide well with previously published examples obtained with other potentiometric multisensors. Meanwhile the ISEs based on combination of polyaniline and macrocyclic receptors exhibit specific properties that affect the behavior of appropriate ET. First, the use of polyaniline as intermediate layer with the electron-to-ion conductivity increases the influence of the sample acidity on the signals especially in neutral and weak acidic media that is typical for many refreshments, fruit juices and vegetable extracts. The pH sensitivity of polyaniline is partially suppressed by the thiacalix[4]arene based ionophores deposited on its surface without any plastic support. This results in smoothen pH dependency of the electrode potential and of the potentiometric selectivity of sensor. Then, most of the sensors described do not assume the use of lipophilic salts and plasticizer due to rather higher plasticity of the macrocycle coverage. This simplifies assembling the ISE by casting the solution of the thiacalix[4]arene in organic solvent and its drying at ambient temperature. The response of such electrodes retains its reversibility in the series of measurements with alternating analyte concentrations. However, the formation of rather thick films of thiacalix[4]arene results in a relatively slow response of about 20 s for 95% shift of the potential.

The idea to use solid-contact potentiometric sensors based on polyaniline and thiacalix[4]arene ionophores assumes the detection of the polyaniline redox status. The interaction of the Fe(III) ions with thiacalix[4]arenes affects their surface concentration and participation in the partial oxidation of polyaniline. All the factors influencing the surface reactions change the stationary potential of the sensors. Such processes can involve:

- Direct influence of the antioxidants on the redox potential of the Fe(III)-polyaniline system;
- Changes in the pH and buffer capacity caused by the sample addition and their influence on the redox equilibria of polyaniline and hydrolytic stability of Fe(III) ions;
- Complexation of the Fe(III) ions with the sample components, e.g., citrates or polyphenolics and flavor additives;
- Adsorption of the lipophilic species on the ISE surface promoted by thiacalix[4]arene bearing long-chain alkyl radicals in the substituents.

Relative contribution of the factors mentioned depends on the analyte and discrimination parameters. Thus, in case of the fruit juices, the influence of antioxidants dominates. This is confirmed by simultaneous measurements of the antioxidant capacity of the samples. For tea infusions, higher importance of complexing agents was mentioned. Regarding the pH and buffer capacity influence, their deviation is often rather narrow within the set of the samples of the same origin, and this complicates assessment of their influence.

The application of the ET systems described requires preliminary study on the optimal number of sensors, sample dilutions, and Fe(III) ion concentration. Although such a choice might be a subject of personal preferences of an analyst, it was established for many experiments among various beverages and foodstuffs that higher difference between the dilutions improves the quality of discrimination. The absolute value of dilution varied from 1:1 to 1:200 depending on the total content of oxidizable species. The latter parameter can be expressed by antioxidant capacity (activity) determined by conventional techniques including coulometry with electrogenerated bromine.

The necessity of the Fe(III) ions for amplifying the signal is related to the S-shape of its calibration curve and hence high sensitivity of the ISE potential to the minor changes in the concentration and accessibility of reactants. In case of beer discrimination, the experiments confirmed the possibility for separation of light and dark beer types with no Fe(III) addition. The same conclusion has been made later for soya sauce discrimination (not published). Besides, similar efficiency of separation was shown for apple juices of different manufacturers with Ag^+^ ions. However, the results were sensitive toward content of chlorides and some other anions able to form insoluble salts with Ag cation.

Simple tuning of the sensor performance is another advantage of the solid-contact sensors described. Increasing amounts of polyaniline deposited by electropolymerization prior to thiacalixarene deposition as well as anodic conditioning of the electrodes improved the redox reversibility and the pH sensitivity of the signal. The slopes of appropriate calibration curves easily control the quality of the modifying layer prior to their application in the ET system. The selectivity of thiacalix[4]arenes in the assembly of the ISEs can be also modified by implementation of appropriate functional groups in the macrocycle moiety or by specifying its configuration. Although no correlation between the steric factors (configuration of thiacalix[4]arenes) and selectivity of the cation binding were found, from the basic point of view, *1,3-alternate* and *partial cone* should have advantage over *cone* configuration due to higher accessibility of the terminal groups and less dependence of recognition on the position of the ionophore molecule on the underlaying surface. The prospects of ET systems are expected to be extended after implementation of pillar[5]arene ionophores that are more sensitive to lipophilic properties of the sample components and factors affecting hydrogen bonds and self-aggregation of the macrocycle molecules.

Most of the results reviewed were obtained with only two derivatives **2** and **3**, which provided cross-selectivity sufficient for the ET operation. Meanwhile other thiacalixarene derivatives were compared in beer testing. Among them, carboxylated and aminated thiacalix[4]arenes with lowest selectivity toward inorganic cations and acidic species were used but not proved to improve the performance of the ET system. Ranking individual sensors in accordance with the efficiency of prediction estimated by PCA or LDA is a promising way to optimize the set of ISEs combined in the ET system. This problem is often underestimated in other multisensors based on conventional ISEs but directly influences the efficiency of application and final cost of the analysis/assessment.

Summarizing the results of assembling of solid-contact sensors with thiacalix[4]arene ionophores and of their application in the ET systems, it can be concluded that this is a cost- and labor-effective way to develop the ETs intended for preliminary assessment of the quality of many products reach with antioxidants and organic acids and demanded in the food industry, environmental monitoring and medicine.

## Author contributions

GE worked under the concept of electronic tongue based on discrimination of various species on the base of their antioxidant properties and elaborated protocols for the assessment of foodstuffs and beverages in the presence of Fe(III) ions. He also performed discrimination of alcoholic drinks; MS performed experiments concerning individual potentiometric selectivity of the ISEs with thiacalix[4]arene and pillar[5]arene ionophores. Besides, he worked with the screen-printed electrodes as transducers and adapted the protocol of polyaniline polymerization to reach reversibility of its characteristics; SB has assembled solid-contact sensors with polyaniline and thiacalix[4]arene ionophores, investigated their operational and analytical characteristics and established optimal conditions for the determination of individual metals and discrimination of the metals in their binary mixtures; IS was responsible for the synthesis of substituted thiacalix[4]arenes and pillar[5]arene and their physicochemical characterization; RS conducted experiments with fruit juices and beer using electronic tongue system based on polyaniline and thiacalix[4]arenes.

### Conflict of interest statement

The authors declare that the research was conducted in the absence of any commercial or financial relationships that could be construed as a potential conflict of interest.
